# A Diagnostic Chip for the Colorimetric Detection of *Legionella pneumophila* in Less than 3 h at the Point of Need

**DOI:** 10.3390/bios14050228

**Published:** 2024-05-04

**Authors:** Katerina Tsougeni, Anastasia Kanioura, Athina S. Kastania, Kosmas Ellinas, Antonios Stellas, Vassilios Constantoudis, Galatios Moschonas, Nikolaos D. Andritsos, Manolis Velonakis, Panagiota S. Petrou, Sotirios E. Kakabakos, Evangelos Gogolides, Angeliki Tserepi

**Affiliations:** 1 Nanoplasmas P.C., “Lefkippos” Technology Park, Patriarchou Gregoriou E’ & 27 Neapoleos Str., P.O. Box 60037, Ag. Paraskevi, 153 41 Athens, Greece; k.tsougeni@nanoplasmas.com (K.T.); a.kastania@inn.demokritos.gr (A.S.K.); k.ellinas@nanoplasmas.com (K.E.); ypetrou@rrp.demokritos.gr (P.S.P.); skakab@rrp.demokritos.gr (S.E.K.); e.gogolides@inn.demokritos.gr (E.G.); 2 National Centre for Scientific Research “Demokritos”, Patriarchou Gregoriou E’ & 27 Neapoleos Str., Ag. Paraskevi, 153 41 Athens, Greece; v.constantoudis@nanometrisis.com; 3 Nanometrisis P.C., “Lefkippos” Technology Park, Patriarchou Gregoriou E’ & 27 Neapoleos Str., P.O. Box 60037, Ag. Paraskevi, 153 41 Athens, Greece; 4 Eurofins Athens Analysis Laboratories, 29 Nafpliou Str., Metamorfosi, 144 52 Athens, Greece; galatios.moschonas@ftcee.eurofins.com (G.M.); nandritsos@upatras.gr (N.D.A.);

**Keywords:** lab-on-a-chip, DNA amplification, colorimetric detection, waterborne bacteria, *L. pneumophila*, capturing antibodies, LAMP amplification, image analysis

## Abstract

*Legionella pneumophila* has been pinpointed by the World Health Organization as the highest health burden of all waterborne pathogens in the European Union and is responsible for many disease outbreaks around the globe. Today, standard analysis methods (based on bacteria culturing onto agar plates) need several days (~12) in specialized analytical laboratories to yield results, not allowing for timely actions to prevent outbreaks. Over the last decades, great efforts have been made to develop more efficient waterborne pathogen diagnostics and faster analysis methods, requiring further advancement of microfluidics and sensors for simple, rapid, accurate, inexpensive, real-time, and on-site methods. Herein, a lab-on-a-chip device integrating sample preparation by accommodating bacteria capture, lysis, and DNA isothermal amplification with fast (less than 3 h) and highly sensitive, colorimetric end-point detection of *L. pneumophila* in water samples is presented, for use at the point of need. The method is based on the selective capture of viable bacteria on on-chip-immobilized and -lyophilized antibodies, lysis, the loop-mediated amplification (LAMP) of DNA, and end-point detection by a color change, observable by the naked eye and semiquantified by computational image analysis. Competitive advantages are demonstrated, such as low reagent consumption, portability and disposability, color change, storage at RT, and compliance with current legislation.

## 1. Introduction

*Legionella pneumophila* has recently been pinpointed by the World Health Organization [1] as the highest health burden of all waterborne pathogens in the European Union, with many outbreaks reported around the globe every year. *Legionella* has been found in a wide range of systems, due to its preference for temperatures between 25 and 55 °C, including hot- and cold-water systems, cooling towers, certain types of air conditioning systems, and generally in various water sources such as showers, swimming pools, and fountains, where *Legionella* can be aerosolized [2]. Infection occurs upon breathing air containing water droplets contaminated with bacteria and is fatal in approximately 10–15% of cases, while this percentage is further increased in patients admitted to intensive care units [3]. As the concentration of *Legionella* bacteria can double every 90 min under favorable conditions, a very low initial bacterial concentration could increase thousands of times in one day [4]. To prevent outbreaks and deaths, the European Centre for Disease Prevention and Control recommends applying regular checks of man-made water systems and taking appropriate control measures to prevent Legionnaires’ disease at recreation centers, accommodation sites, hospitals, or other settings, where sizeable populations may be exposed [5]. The most established method for bacteria detection is based on bacteria culturing onto agar plates (ISO 11731 [6] for *Legionella*). However, this method is characterized by limited sensitivity, a long incubation period (approximately 12 days for *Legionella*), false-negative results, and an inability to be implemented for fast and accurate analysis or for multiple-sample analysis in facilities of public interest [7]. 

Towards this direction, novel methods have been developed to provide results in less than two days. These methods include immunological methods, flow cytometry, optical and electrochemical biosensors, gas sensors, and microfluidic platforms [8,9,10,11]. Nevertheless, two of the most challenging issues, sample preparation/preconcentration and sensitive detection in a portable format, still remain unresolved. Therefore, compact yet simple, rapid, accurate, cost-efficient, real-time, and user-friendly methods are still needed for the on-site detection of waterborne pathogens. This requires further development and tailoring of revolutionary technologies, and this is the gap that the present work promises to fill.

The advent of microfluidics and laboratories on a chip (LoC), enabling the on-chip integration of all steps from sample preparation to detection, brings many advantages, such as reduced time, smaller sample and reagent volumes, low contamination risk, automation, multiple and parallel sample detection, low cost, and compatibility with downstream processes [12,13,14,15]. Additionally, all analytical steps, such as sample pre-treatment, chemical reactions, and on-line quantification, can be integrated into a single, portable microfluidic platform for on-site testing applications [16,17,18,19]. However, the detection of waterborne bacteria by microfluidics-based methods is still challenging due to the inherent limitations related to the use of small sample volumes in microchannels, presenting obstacles in terms of desirable sensitivity, selectivity, and stability for microfluidic sensors.

The scientific community has proposed portable micro/nano/biosystem prototypes with distinct chip designs for the ultra-fast analysis of pathogens in food, accommodating bacteria capturing and lysis as well as DNA amplification and detection, to cope with the urgent problem of foodborne outbreaks [20,21,22]. Among various DNA amplification techniques, LAMP is considered advantageous due to its fast amplification, simple operation at a single temperature, robustness, tolerance to inhibitors, and high sensitivity and specificity. The specificity of LAMP is higher than that of PCR, thanks to the use of four to six different primers that specify the template strand to be amplified. LAMP amplifies nucleic acid at temperatures between 60 and 66 °C by leveraging the Bst polymerase enzyme with high strand displacement activity [23,24], and thus, in contrast to PCR, it is able to perform the test in the absence of any sophisticated equipment. One limitation of LAMP for use at the point of interest is the need for a refrigerator for the storage of the reagents. For this reason, the lyophilization of LAMP reagents and their stability have been studied in order to simplify the analysis for the user. Lyophilization has been successfully reported with the addition of sugars in the reaction mixture for the detection of bacteria [25,26,27,28] and viruses [29,30]. Lyophilized samples are usually stored in aluminum pouches with silica gel at temperatures varying from −20 °C to 37 °C. In previous research, when stored at 37 °C, lyophilized reagents were found to be stable for a maximum of 14 days [25,26,27,28]; at room temperature (25 °C), they were stable for almost a month [25,26,27,28,29]; and at lower temperatures (4 °C, −20 °C), they remained stable from 30 to even 720 days [26,27,28].

In integrated LoC platforms, an on-chip sample preparation module is typically connected to a sensing area or a sensor housed on the same substrate or heterogeneously integrated. There are several detection schemes and sensors, such as electrochemical, capacitive, conductivity, and several optical ones, based on fluorescence, turbidity, and colorimetry. Colorimetric methods are easy to use, and, often, detection can be achieved by the naked eye in the form of a color change. Typically, such methods are based on fluorescence enhancement by nanoparticles in order to be easily observable by the naked eye [31]. Rarely, a simple color change may also be used; however, in this case, a significant optical path is needed (in the millimeter range), which is not easily realized in a microfluidic chip (a few tens to a few hundreds of microns deep). Hence, typically, samples are directed to some type of well or large microreactor, where the depth is enough to allow optical observation [32,33]. Therefore, sample preparation is rarely achieved in the same microfluidic channel as the detection via a color change. With the exception of our previous work [22], there are no approaches where sample preparation and DNA amplification are both accommodated on the same microfluidic chip, offering a small footprint, user friendliness, and high specificity.

With the significant development of various LoC devices, several LAMP-based diagnostic platforms have been developed for the detection of *Legionella* and other bacteria [34,35,36,37,38,39] and have been commercialized [40,41,42,43,44,45,46]. The detection is usually based on fluorescence or turbidity measurements; the cost per test varies from USD 2 to 80, while the cost of the accompanying reader ranges from USD 2.5K to 25K. Significant features are the detection time that ranges from 20 min to 6 h and the limit of detection (LOD) that ranges from 10^2^ to 10^5^ CFU/mL [40,41,42,43,44,45,46]. The most significant shortcoming is that all of these commercial platforms accommodate DNA amplification only, while most of the steps for sample preparation are performed externally. 

The present work provides a method for the rapid diagnosis of *Legionella* spp., in particular of *L. pneumophila*, in less than 3 h (instead of the 12 days required in the ISO method [6,7]) by developing an optimized LoC for rapid, sensitive, specific, and precise colorimetric end-point detection on a chip. This chip is the first to integrate sample preparation and detection in the same microchannel. In addition, the described diagnostic LoC exhibits certain advantages over standard technologies by achieving the following: a reduction in analysis time by 99%; a simplification of fabrication by using a widespread manufacturing technology (i.e., machining); cost-effectiveness using simple bio-processes and small reagent volumes; and a generic product able to be used in many applications based on bacterial DNA detection. The potential impact of this diagnostic chip is huge as governments attempt to tackle the worldwide spread of infectious diseases. In fact, the same technology with the appropriate choice of antibodies and DNA amplification primers can be used for the detection of any pathogenic bacteria other than *Legionella*.

## 2. Materials and Methods

### 2.1. Materials

Clear plexiglass sheets, 4 mm thick, were purchased from IRPEN (Spain).

Plasma treatment was performed in a parallel-plate reactive-ion etcher (from Nextral, France) operating at 13.56 MHz. PMMA samples were treated, typically for 12 min, in O_2_ plasma under highly anisotropic etching conditions (O_2_ flow: 100 sccm; plasma power: 400 W; pressure: 10 mTorr). After the plasma treatment, an annealing step was performed in an oven at 100 °C for 2 h (i.e., below the glass transition temperature, T_g_ = 109 °C, of the polymer).

The chip was sealed with lamination using pressure-sensitive adhesive tape (3M Advanced Polyolefin Microplate Sealing Tape 9795 from 3Μ Co, Olitecn Lab Equipment, Athens, Greece). 

Anti-*Legionella pneumophila* rabbit polyclonal antibody was purchased from VWR International, LLC (Radnor PA, USA). Lyophilization (freeze drying) was performed using the FDL-10N-50-TD system from MRC Lab (Harlow, UK). 

Certified reference material (CRM) of *L. pneumophila* (CRM12821M LENTICULE^®^ discs, Supelco (Bellefonte, PA, USA)) was purchased from LIFESCIENCE (Athens, Greece) and kept at −20 °C until used. Ringer’s solution was purchased from MERCK (Darmstadt, Germany). A Triton mix (Triton X-100), purchased from New England Biolabs (NEB, Ipswich, MA, USA), was used as lysis buffer. The WarmStart Colorimetric LAMP 2X Master Mix (M1800 or M1804S with UDG) LAMP amplification kit and DNAse- and RNAse-free water were purchased from New England Biolabs (NEB, Ipswich, MA, USA).

### 2.2. Bacteria Cultures and Counting

Once removed from the freezer, the LENTICULE^®^ discs were transferred to ambient temperature within 1 h. The CRMs were then rehydrated in 10 mL of 1/40 Ringer’s solution for approximately 10 min; appropriate serial dilutions were conducted in the same liquid media; then, 0.1 mL of the diluted CRMs was surface spread-plated over glycine vancomycin polymyxin B cycloheximide (GVPC) (VWR, Radnor, PA, USA) plates. Inoculated plates were incubated at 36 °C for 8–10 days and the number of colonies obtained was counted.

### 2.3. Chip Design and Fabrication

**Figure 1:**
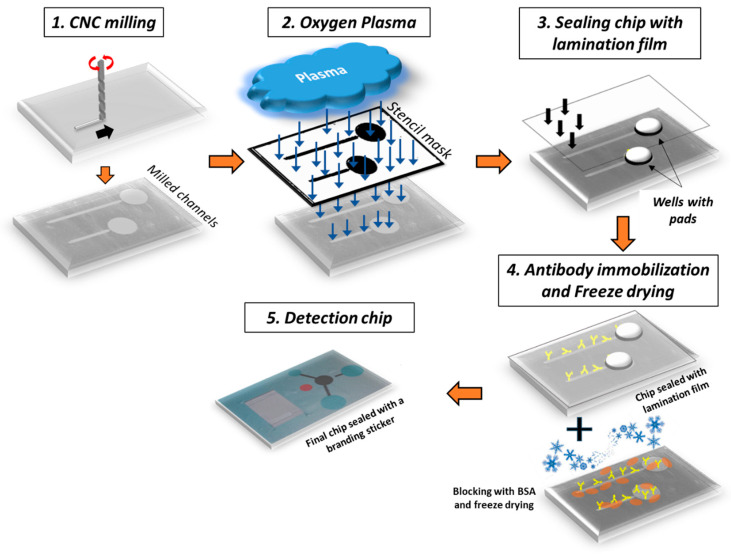
Process flow for the fabrication of the diagnostic lab-on-a-chip for water analysis. An optical image of the chip composed of 2 microchannels with immobilized antibodies (step 4) for hosting negative (i.e., no cells or DNA template present) and positive samples is also shown (step 5). Microchannels’ depth is ~500 μm, while roughness height on PMMA microchannel bottom after O_2_ plasma etching is ~5 μm.

The chip comprised two straight microfluidic channels, each connected to an inlet and a waste well (see Figure 1). One microchannel was used as a control and the other as a sample microchannel (17 mm long). A superhydrophobic valve connected the microfluidic channel to the waste well to prevent sample loss or waste backflow. In detail, a constriction of the microchannel width down to 500 μm was fabricated by CNC milling and was subsequently coated by drop-casting a commercial hydrophobic thin film (Teflon^®^ AF 1600 from DuPont) to act as a superhydrophobic valve [47,48] that facilitated the complete filling of the microchannel with a liquid volume equal to 25 and 12 μL for the sample and negative control line, respectively. To push liquid to the waste well, new liquid or air should be injected into the microchannel with pressure exceeding the valve operation threshold. The chip was fabricated on a polymeric poly(methyl methacrylate) (PMMA) substrate. Alternative polymers that have been tested include Cyclo Olefin (COP), PS, and PEEK. The process for the fabrication of the chips is shown schematically in Figure 1. The microchannels and wells were fabricated by Computer Numerical Control (CNC) milling (step 1). After CNC, the chip was micro/nanotextured by treatment (through a stencil mask) with O_2_ plasma (step 2; for processing details, see Section 2.1). The exposed chip surface was etched and simultaneously nanotextured to acquire a high surface area [49,50,51,52]. Subsequently, the microchannels and waste wells were sealed (step 3) with a pressure-sensitive adhesive film (in a laminator). Following sealing, the capturing antibodies (at a high concentration) were immobilized on the micro/nanotextured surface (step 4) to achieve bacteria capturing and isolation with an extremely high specificity and efficiency. It was proven convenient to lyophilize the antibody solution added into the microchannels (step 4) to allow the long-term usage of the chips (up to 18 months), facilitate their transport at ambient temperature, and simplify the process for the end-user.

### 2.4. Lyophilization (Freeze-Drying) Process for L. pneumophila Antibody on Chip

To enable the easy storage of the chips, the antibodies were immobilized and the functionalized surface was subjected to freeze drying in the presence of BSA [21,22]. The process followed is described in detail below:

*L. pneumophila* antibodies (4 mg/mL stock initial solution) were immobilized onto the sealed chips through the injection of 25 μL of a 100 μg/mL solution in carbonate buffer (pH 9.2, 50 mM) and overnight incubation at 4–8 °C in a controlled-humidity chamber (75% humidity). Then, the washing of the chip surfaces with 25 μL of 10 mM Tris-HCl, pH 8.25, and 9 g/L NaCl (washing buffer) and blocking using 25 μL of the 10 g/L BSA solution in 0.1 M NaHCO_3_, pH 8.5, for 1 h at RT followed. After blocking, cooling down to around −50 °C took place by lowering the freeze-dryer shelf temperature for approximately 5 h to ensure uniform supercooling across the batch. The antibodies were incubated in the chip overnight (~18 h). Following freeze drying, the chips were stored inside Mylar foil pouches together with desiccant bags and kept at room temperature (RT) until use (for up to 18 months without losing their functionality).

### 2.5. Sample Preconcentration and Chip Operation

**Figure 2:**
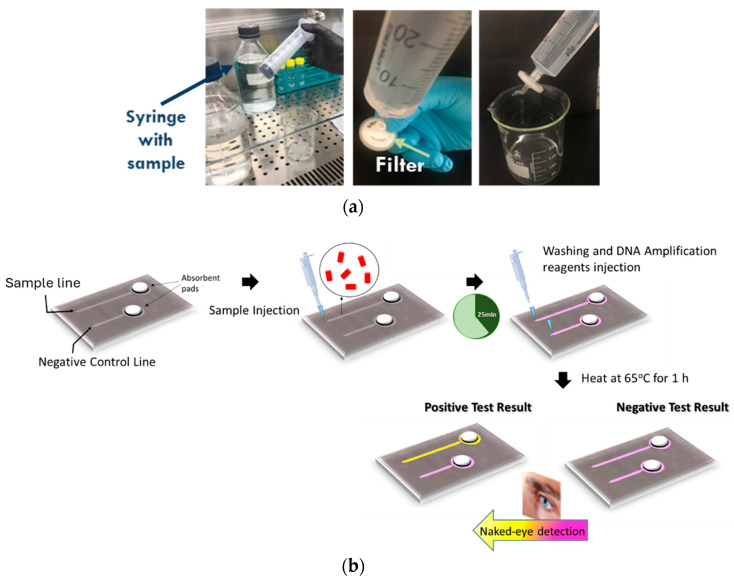
The two-step detection protocol for *Legionella* included (**a**) a short (5 min) off-chip filtration step for sample preconcentration, starting from 200 mL of artificially contaminated water, and (**b**) on-chip bacteria capture, lysis, DNA amplification, and color change (naked eye) end-point detection.

Sample preconcentration is necessary in order to reduce the water sample volume from several hundreds of milliliters to a few hundred microliters. For this reason, a filtration-based sample preconcentration protocol was developed (Figure 2a). Two hundred milliliters of artificially contaminated water or another liquid sample was filtered using 0.2 μm pore size filters. The bacterial pellet that formed on the filter was resuspended in 200 μL of buffered solution. 

Subsequently, 100 μL of the resuspended pellet solution was injected into the functionalized chip to initiate antibody-based bacteria capture (Figure 2b). Following bacteria capture, a lysis buffer and a LAMP amplification cocktail were introduced. In detail, the chip operation sequence after bacteria capturing was as follows: PBS buffer injection to wash the unbound bacteria (total volume: 30 μL), LAMP reagent injection (total volume: 24 μL), chemical lysis with triton (included in the LAMP reaction) for 10 min, and chip heating on a plate at 65 °C for up to 60 min. DNA detection can be achieved visually by detecting the color change by the naked eye or by a camera. 

For increased capturing efficiency, the chip was operated in batch mode as follows: A sample volume equal to the microchannel volume (25 μL) was introduced and was allowed to react with the antibodies for an optimized time of 25 min. Then, the sample was pushed to the waste well and a second batch of equal volume was allowed to enter and react with the antibodies. Following bacteria capture on the microchannel surface from 4 batch samples, in situ lysis and specific DNA amplification were performed, followed by chromogenic naked-eye end-point detection to allow us to reach a decision on the potential presence of living bacteria with high sensitivity. 

### 2.6. Amplification Protocol for on-Chip Detection of L. pneumophila

The sequences of the primers used in this work for DNA amplification via LAMP were as follows:

F3_CGTTACCCACAGAAGAAGC;
B3_ACCCTCTCCCATACTCGA;
FIP_AGTAATTCCGATTAACGCTCGCAACCGGCTAACT CCGTGC;
BIP_GGCGTAAAGGGTGCGTAGGTGACCAGTATTATCT GACCGTCC.

The LAMP assay for *Legionella* detection is shown below:

The LAMP reagent mix, with a total volume of 25 μL, contained the following: 12.5 μL of WarmStart Colorimetric Lamp 2X Master Mix (New England BioLabs, Ipswich, MA, USA), 2.5 μL of 10× primer mix (16 μM (each) of Forward Inner Primer (FIP) and Backward Inner Primer (BIP); 2 μM (each) of F3 and B3 primers), 8 μL of DNAse- and RNAse-free water, and 1 μL of Triton mix (10% Triton X-100 + 44 μL of DNAse-free water + 0.5 μL of 1 M Tris buffer pH 8.4 + 0.5 μL of 5% phenol red solution) for chemical lysis and 1 μL of any crude water sample, containing cells or not. After 5–10 min of waiting for the chemical lysis with Triton X-100 to take place, the chip was placed on a heating plate at 66 °C for up to 60 min. Amplification in the presence of target nucleic acids results in the production of protons that decrease the mixture’s pH, resulting in a color change in the phenol red from pink to yellow that is easily detectable by the naked eye. For validating amplification, the DNA amplicons were analyzed using electrophoresis on 2% agarose gel containing GelRed (Biotium, Lab Supplies, Athens, Greece) and visualized under UV light.

### 2.7. Lyophilization Process (Freeze Drying) for the Amplification Cocktail for L. pneumophila

To facilitate the use of the amplification cocktail outside analytical laboratories, i.e., at the point of need or in resource-limited settings, the typical requirement for the storage of the reaction mixture components at −20 °C should be lifted. Thus, to allow room-temperature storage and create a stabilized form of the reaction mixture, lyophilization of the LAMP reagents for the detection of *Legionella* spp. was attempted. 

The addition of cryoprotective substances was investigated, as proposed by Klatser et al. [53] and Agel et al. [54]. In this work, it was found that the addition of the non-reducing sugar D-trehalose together with a small concentration of BSA stabilized the reagents during freeze drying. After lyophilization, the amplification cocktail was stored inside Mylar foil pouches together with desiccant bags. The Mylar foil pouches acted as an effective barrier against air moisture with very low water vapor transmission rates, oxygen, and light, resulting in a more stable dehydrated reagent mixture. The lyophilized reaction mixtures in the presence of D-trehalose/BSA, stored inside Mylar foil pouches, retained their activity for prolonged periods, i.e., more than 3 months under dry conditions at room temperature, without compromising the performance of the amplification cocktail.

### 2.8. Optical Image Analysis

In order to achieve a robust diagnosis for the presence of bacteria in the sample based on the color change in the microchannel, automated detection of the color change was attempted. To this end, a computational methodology was designed and realized in a software application, based on the analysis of camera photos of the chip, targeting an assessment of the color changes of the negative control and (sample) detection channels. The proposed methodology consisted of three steps following the acquisition of a photo of the chip after sample analysis. First, the automatic identification and isolation of the regions of interest of the chip photo, i.e., of the control and detection channels, were performed. Second, the color change in these areas (channels) was quantified through the measurement of a specific color index. Third, using standard samples of known bacterial concentrations, the proposed color index was assessed as a robust means on which the diagnosis result can be based.

**Figure 3:**
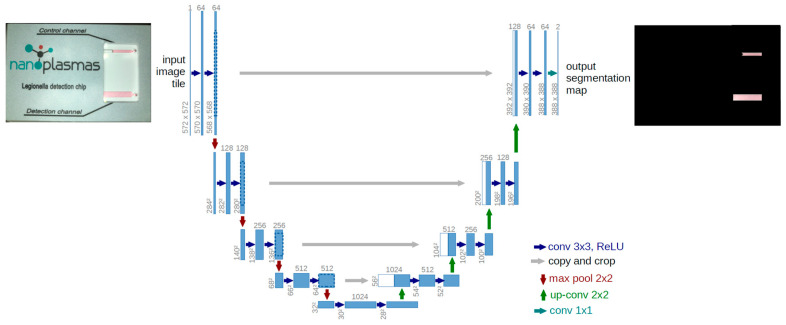
Architecture of a U-Net convolutional neural network architecture along with an example of an input chip photo and the output with highlighted identified channel regions.

1st step: To achieve the isolation of the detection and control channels, a machine learning model architecture, called U-Net [55], was trained using images from samples analyzed on chip. U-Net is a convolutional neural network architecture developed for image processing and object recognition applications. It consists of an “encoder” and a “decoder” that are combined with a connection bridge. The encoder detects high-level features in the image, while the decoder reconstructs an exact match of the original image. This architecture, shown schematically in Figure 3, is highly efficient for tasks such as the requirement herein, namely the isolation of objects in an image (i.e., the detection and control channels). It must be emphasized that U-net produces a “segmentation map”; i.e., it produces an image with the same dimensions but with active pixels only in the areas of interest (control and detection channels). Therefore, by knowing the positions of the active pixels in the result, we can locate the corresponding positions in the original image.

2nd step: The color change was analyzed for each isolated channel. The digital quantification of a color involves the use of a model, such as the Hue Saturation Value (HSV) system. A colored image consists of a collection of pixels, with each pixel containing a set of three HSV coordinates. In our software, we used the hue value of the HSV model to quantify the channel color and, thus, based on its change, whether the sample was positive or negative. 

3rd step: The assessment of the proposed hue value was carried out using samples of known bacterial concentrations, as will be shown in Section 3.7. 

## 3. Results

### 3.1. Sample Preconcentration

**Figure 4:**
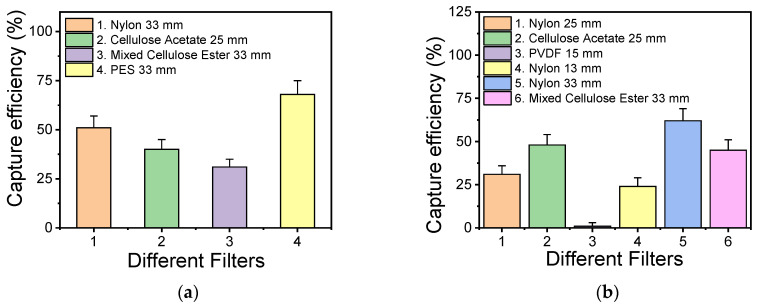
Efficiency of various commercial filters with 0.2 μm pore size for capturing *L. pneumophila* (**a**) and *Salmonella* (**b**) bacteria in spiked water samples.

Filtration-based sample preconcentration was implemented to reduce the sample volume from 200 mL to 100 μL, which is easily handled in the microfluidic channel. Filters from different materials were tested, all with a 0.2 μm pore size. To assess each filter’s capturing efficiency, the solution resuspended from the filter was cultured in plates, and the viable bacteria colonies were counted and compared to the original bacterial concentration spiked in the water sample. For comparison purposes, *Salmonella* bacteria were also used, in addition to *L. pneumophila*. In Figure 4, the capturing efficiency for *L. pneumophila* and *Salmonella* is shown for different filters. Figure 4a shows the capturing efficiency for *L. pneumophila,* upon the filtration of 5 × 10^3^ CFU/mL of bacteria, while Figure 4b shows the capturing efficiency for *Salmonella* when filtering a similar bacterial concentration on different filters. It can be observed that for *L. pneumophila*, the best working filters (with a capturing efficiency close to or higher than 50%) are poly(ether-sulfone) (PES) and nylon, while for *Salmonella*, the best working filters are nylon, cellulose acetate, and cellulose ester.

### 3.2. Bacteria Capturing

**Figure 5:**
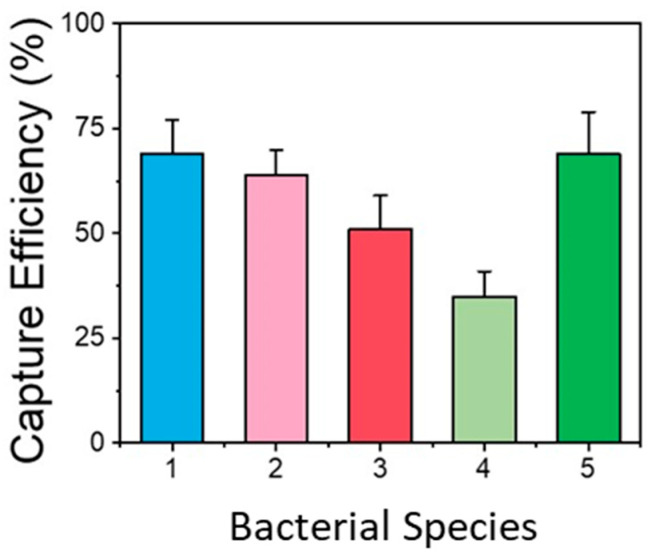
Capturing efficiency of functionalized micro/nanotextured microchannel for different bacteria (1: *E. coli*, 2: *Salmonella*, 3: *Listeria*, 4: *B. cereus*, 5: *L. pneumophila*). The results obtained were very repeatable, and all the values were within ±15% of the mean value obtained for each bacterial species.

Resuspended typical water sample volumes of 150–200 μL, equal to the filter volume, were used for increased detection sensitivity. However, flowing the water sample continuously through the chip may significantly reduce the bacteria capture efficiency. Therefore, the chip was operated in batch mode as follows: A sample volume equal to the microchannel volume (25 μL) was introduced and was allowed to react with the antibodies for a time of 25 min for enhanced bacteria capturing. Then, the sample was pushed to the waste well and a second batch of equal volume was allowed to enter and react with the antibodies. The sample introduction in four batches maintained the high efficiency of the chip while keeping the chip volume as low as possible and yet compatible with the volume of the amplification kit. The capturing efficiency of the chip was assessed for five different bacteria (*L. pneumophila*, *E-coli*, *Salmonella*, *Listeria*, and *B. cereus*), with the sample directly injected into the chip in four batches (as described above), after comparing the bacterial concentration exiting the micro/nanotextured microchannel that had been functionalized with the respective bacteria-specific antibodies (with an injected bacterial concentration of 200 CFU/100 μL). The results are shown in Figure 5. The capture efficiency depended on the bacterial species and was between ~35% and 70% (the highest efficiency was observed for *E. coli*, *Salmonella*, and *L. pneumophila*).

### 3.3. Optimization of Microchannel Depth for LAMP-Based DNA Detection

**Figure 6:**
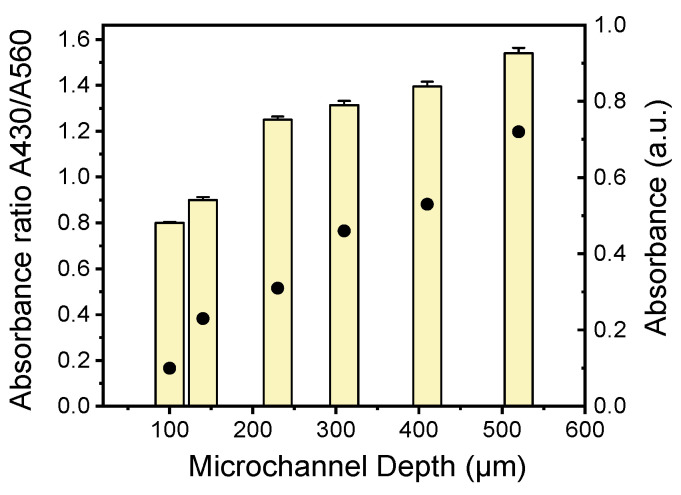
Absorbance (dots) and absorbance ratio (bars) of A430 to A560 nm of LAMP-amplified *Legionella* DNA in PMMA microchannels of various depths.

In this work, among the different isothermal DNA amplification techniques, LAMP was chosen due to its utmost benefits (simple operation, robustness, and high sensitivity and specificity). Nucleotide incorporation by the DNA polymerase during amplification releases protons, changing the color of the pH-sensitive dye, phenol red, contained in the reaction mix. Since bacterial LAMP-based DNA detection is color-based, the microchannel depth should be optimized to achieve sufficient visible-light absorbance. To provide quantitative results, the absorbance at 430 nm and 560 nm was assessed by means of a UV–visible spectrophotometer (from θ-metrisis, Athens, Greece) equipped with a CCD detector, and the reaction performance was measured by the change in the ratio of light absorbed at 430 and 560 nm. End-point absorbance measurements of the LAMP-amplified DNA in the microchannels were performed for microchannels of increasing depth (from 100 to 140, 230, 310, 410, and 515 μm), and the absorbance ratio A430/A560 was calculated for all microchannel depths. The absorbance ratio A430/A560, which is proportional to the drop in pH occurring by the production of protons as the LAMP amplification progresses, is shown in Figure 6 as a function of the microchannel depth. As expected, the absorbance ratio A430/A560 increases as the microchannel depth increases. Thus, a microchannel depth of 500 μm was chosen as the depth sufficient to achieve a high absorbance ratio that can be easily manufactured by CNC in typical PMMA sheets. 

The chip design includes several innovations. As was shown in Section 3.2 and herein, the microchannel’s geometrical characteristics were chosen to maximize the bacteria-capturing surface (microchannel length and roughness) and achieve high-contrast colorimetric detection (microchannel depth), while at the same time keeping the microchannel volume at 25 μL, for a reasonably low reagent volume and operation cost.

### 3.4. Validation of the LoC for Water Sample Analysis

The proposed method for *Legionella* detection combines on-chip bacteria immunoaffinity capturing, chemical lysis, and DNA isothermal amplification, with all three steps accommodated in a single microfluidic chip, followed by simple color change detection in the presence of *Legionella* bacterial DNA in the sample. As explained in Section 2.4, after bacteria capturing on the microchannel wall, the on-chip lysis of captured cells (10 min) and on-chip LAMP-based DNA amplification (65 °C for up to 60 min) followed. The outcome of the amplification reaction based on colorimetric LAMP is observable by the naked eye for detecting the potential color change from pink (negative) to yellow (positive) in the case of bacterial presence in the water sample.

**Figure 7:**
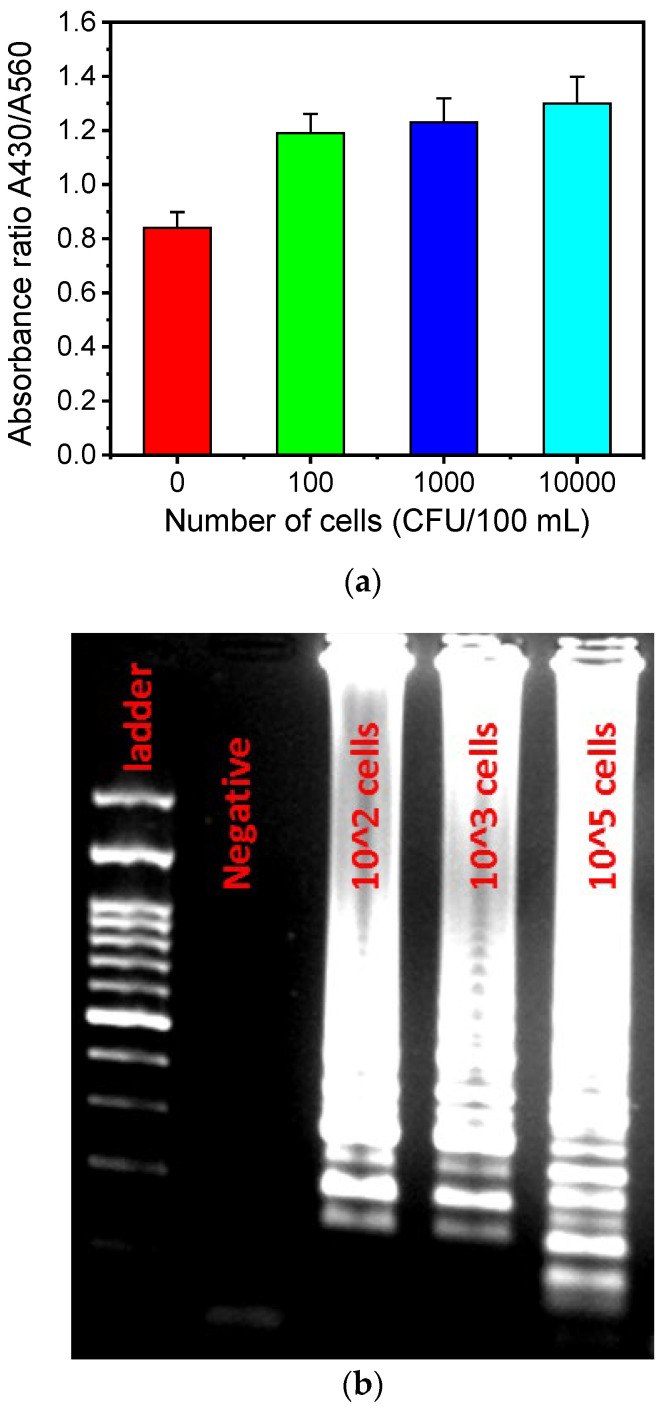
(**a**) Absorbance ratio A430/A560 for various cell concentrations after on-chip colorimetric LAMP amplification. (**b**) Agarose gel image of the colorimetric LAMP amplicons for various *L. pneumophila* bacterial concentrations (CFU/100 mL).

To demonstrate that the LoC can operate with samples of different bacterial concentrations, absorbance measurements were performed for bacterial concentrations in the range of 0–10^4^ CFU/100 mL in artificially contaminated water samples (from an *L. pneumophila* lenticule with standard concentration of 8.6 × 10^3^ CFU), after on-chip capturing and LAMP amplification (Figure 7a). The absorbance, which is proportional to the pH reduction occurring due to the protons produced as the LAMP amplification progresses, increases significantly (by 50%) for bacterial concentrations between 0 (negative) and 100 CFU/100 mL, after which the absorbance increase is less prominent. The results were validated with gel electrophoresis (Figure 7b), indicating the sensitive detection of *Legionella*, even at concentrations as small as 100 CFU/100 mL (the *Legionella* concentration limit imposed by the new European legislation [56]).

**Figure 8:**
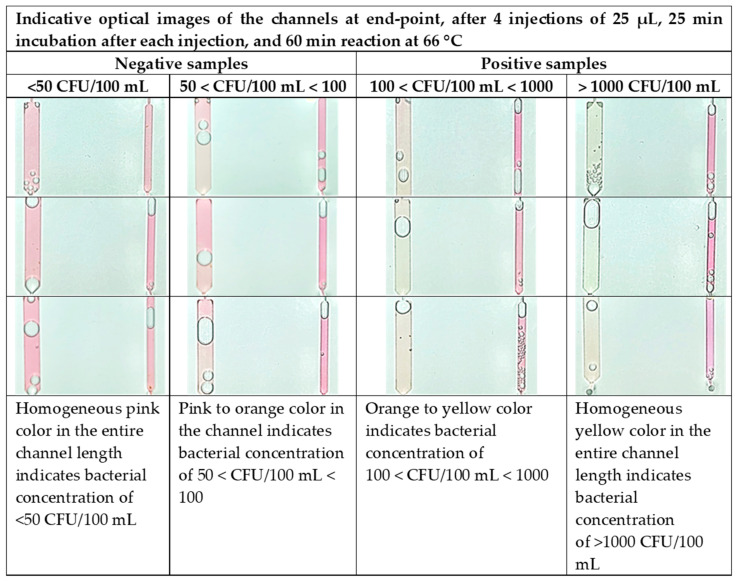
Color code for semiquantification of *L. pneumophila* present in the sample. The chip operation includes 4 water sample injections (25 μL) per sample with incubation of 25 min after each injection, and 60 min LAMP reaction time at 66 °C.

A similar trend was expected for the chip performance assessed by optical images of the chip at the end-point (Figure 8) using artificially contaminated water with different bacterial concentrations, below and above the *Legionella* concentration limit imposed by the new EU legislation (100 CFU/100 mL). The presence of bacterial DNA caused the “sample” channel to turn yellow in color. A “negative control” channel was included, which always remains pink upon successful completion of the test. The images for the “negative” samples (below 100 CFU/100 mL) indicate a homogeneous pink color (<50 CFU/100 mL) or an inhomogeneous pink with orange areas (50 < CFU/100 mL < 100). The images for the “positive” samples show a color shift from faint yellow (for 100 < CFU/100 mL < 1000) to bright yellow (for >1000 CFU/100 mL) for increasing cell concentrations up to 10^4^ CFU/100 mL. After 10^4^ CFU, no significant color difference was observed, indicating saturation in absorbance, in agreement with Figure 7a.

The large color difference between the negative (<100 CFU/100 mL) and positive samples (>100 CFU/100 mL) ensures very sensitive naked-eye detection down to a few tens of cells. We note that the lowest detection limit for on-chip capturing and DNA amplification followed by color detection is between 50 and 100 CFU/100 mL, lower than the *Legionella* spp. concentration limit defined by the existing legislation for drinkable water (100 CFU/100 mL [56]). 

The analytical performance of the *Legionella* detection chip was assessed using two types of samples: water samples spiked with *L. pneumophila* lenticule CRM12821M (standard concentration: 8.6 × 10^3^ CFU/lenticule) and real water samples provided from Athens Analysis Laboratories. For the spiked samples, a total of 130 samples were examined, with concentrations ranging from <50 CFU/100 mL to >1000 CFU/100 mL, created by dilutions of the aforementioned lenticule in 1000 mL. A direct comparison with ISO 11731:2017 [6] showed 100% specificity (95% confidence interval, 99.1 to 100) and 99% sensitivity (95% confidence interval, 98.1 to 99.9). For the real water samples, a total of 111 samples were examined, collected from various hot- and cold-water distribution systems including water basins, taps (including potable water), showers, and water features/swimming pools. A direct comparison with ISO 11731:2017 showed 99.95% specificity (95% confidence interval, 98.07 to 99.93) and 97.9% sensitivity (95% confidence interval, 96.97 to 98.83). 

### 3.5. Reproducibility

**Figure 9:**
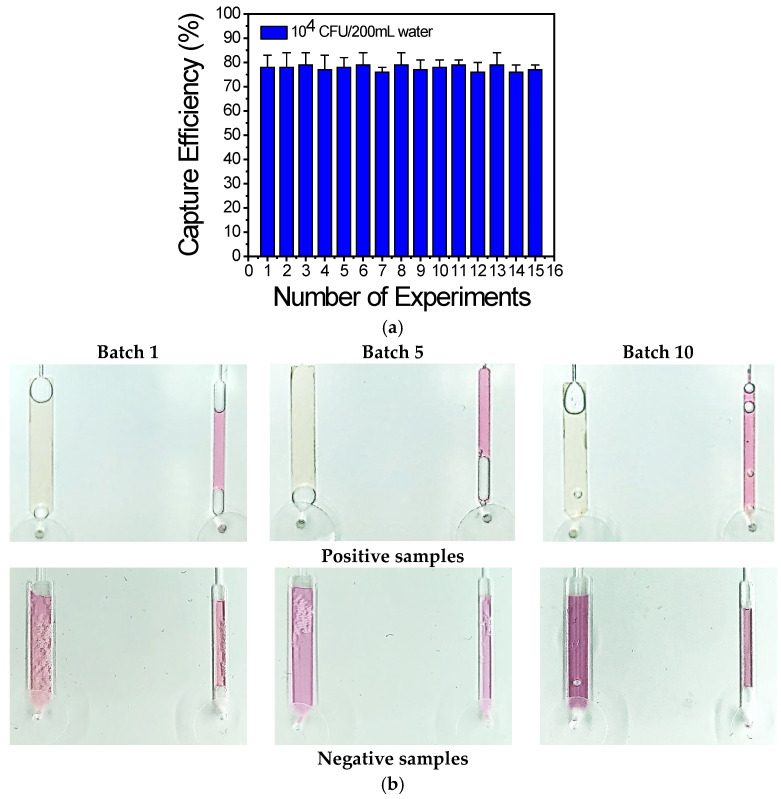
(**a**) Capture efficiency of 15 chips calculated from plating on chip effluents: the capture efficiency is ~80% for experiments carried out over a period of 1 year. (**b**) Representative images of the chips using the optimized assay, obtained from 10 batches of chips prepared and tested over a period of 1 year.

To demonstrate the reproducibility of the on-chip bacteria capture directly from the water samples after preconcentration, *L. pneumophila* capturing was demonstrated directly from the spiked water samples injected into the chip. Figure 9a depicts the capture efficiency (on freeze-dried antibodies) calculated after the plating of effluents coming out of the chip from experiments performed over a period of one year. The capture efficiency was stable over the testing period and equal to ~78% for injections corresponding to ~5 × 10^3^ CFU/100 mL.

To demonstrate the reproducibility of the chip regarding LAMP amplification and visualization, 10 batches of chips were fabricated over a period of 1 year and three chips from each batch were used each time. Representative images of the chip with positive and negative water samples are shown in Figure 9b and indicate reproducible chip fabrication and DNA amplification, as demonstrated by the similar color change or no change for the positive and negative samples, respectively.

### 3.6. The Sensitivity of the Diagnostic Chip for L. pnemophilla Detection

The performance of the method described herein is compared with the reference method ISO 11731:2017, which constitutes the gold-standard method used for the detection of Legionella. In detail, an experimental process was designed based on ISO 16140-4 [57] factorial analysis. Specifically, three types of water samples (i.e., drinking water, non-drinking water, and drilling water) with known contamination concentrations, corresponding to three different levels of Legionella concentration, were used: a zero level (L_0_, do not contain Legionella), medium level (L_1_ > 1–2 × 10^2^ CFU/100 mL), and high level (L_2_ > 2 × 10^3^ CFU/100 mL). In each case, 15 repetitive analyses of each sample were performed. Representative images of the results are shown in Table S1 (in the Supplementary Materials). The zero-level (L_0_) samples were tested to demonstrate that there were no false-positive results (cross-reactivity, e.g., with the water sample). 

The results from the chip-based method described herein and ISO 11731:2017 were in full agreement, demonstrating the good correlation of this method with the gold-standard method. The data were also used to calculate the sensitivity of the diagnostic chip, that is, the probability of a true-positive result concerning the detection performance of the chip, for these three types of water in three different concentrations. The sensitivity data with a 95% confidence interval are shown in Table 1. The data indicate the highest sensitivity (100%) for *L. pneumophila* detection in drinking water, lower sensitivity for drilling water, and even lower sensitivity (although higher than 67%) for non-drinking water. 

### 3.7. Computational Image Analysis

**Figure 10:**
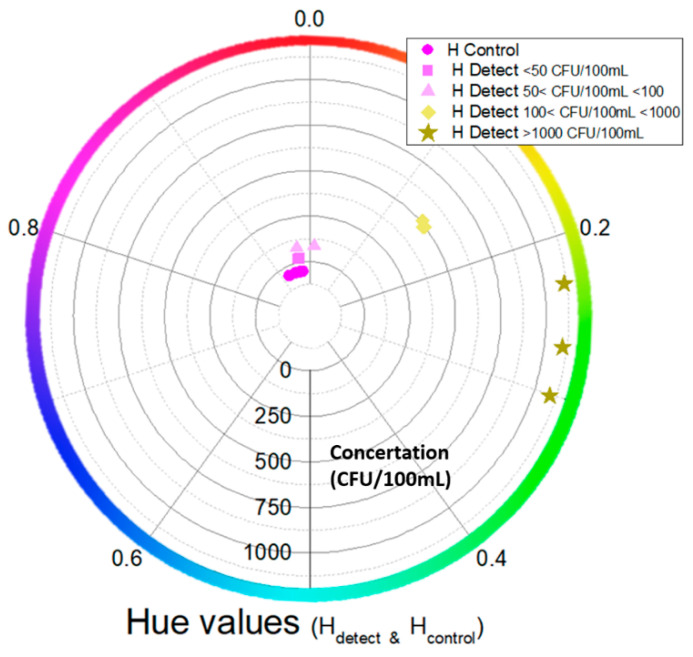
Circular graph of hue values for positive and negative (sample) channels as well as negative control channels vs. bacterial concentrations (from 0 to >1000 CFU/100 mL). Each color and shape (apart from the negative control channel) represent a different concentration (indicated by the concentric circle scale).

Computational color analysis of the camera images such as those in Figure 8 was performed to provide metrics for a robust quantification of the colors in the negative control and (sample) detection channels of the chip. A Hue Saturation Value (HSV)-based methodology was used to quantify the color of both channels (H_detect_ and H_control_) and visualize (as in Figure 10) the classified outcomes based on their color values and their corresponding bacterial concentration. Then, the result was compared with the actual labels to validate the success of the on-chip measurements. In Figure 10, the hue values for the (sample) detection channels (positive and negative) as well as for the negative control channels are shown.

The positive samples (>100 CFU/100 mL) contain hue values higher than 0.1, while the negative ones are between 0.9 and 1/0. The negative samples specifically show hue values close to the control hue values (with overlaps in the hue values close to ~0.93). Also, the negative samples with a concentration of 50 < CFU/100 mL < 100 are close to the negative ones with concentration values of <50 CFU/100 mL. Lastly, we see that the positive samples with concentrations of 100 < CFU/100 mL < 1000 and >1000 CFU/100 mL have a hue value difference larger than 0.05. Therefore, the proposed computational image analysis can provide robust semiquantitative sample analysis based on the hue value.

## 4. Discussion

In the present study, an easy-to-use, compact chip integrating bacteria capturing, lysis, and DNA amplification, all in one microchannel, was developed for the colorimetric, naked-eye, semiquantitative (POS/NEG, based on a color code) detection of *Legionella* present in water samples. A rapid preconcentration (filtering) step was also developed and the optimum commercial filter material (PES) was decided for *Legionella*, demonstrating a capturing efficiency of 70%. Following this off-chip step, a concentrated sample volume (100 μL) was injected into the chip in four batches to accommodate the small chip volume (25 μL). Each sample batch was incubated for 25 min in the chip for the optimum capturing efficiency of *Legionella* (78%) on the functionalized plasma micro/nanotextured PMMA walls of the microchannel. To allow the convenient long-term storage of the chips (up to 18 months), facilitate their transport at ambient temperature, and simplify the process for the end-user, the antibodies immobilized onto the microchannel were lyophilized, thus providing stable-over-time functionality. Following bacteria capturing, the LAMP amplification mixture, containing a lysis buffer, was injected and incubated in the microchannel for 10 min before the chip was placed on a hot plate (heated to 66 °C) for a maximum time of 60 min to complete bacterial DNA amplification and naked-eye detection, thanks to a color change from pink to yellow in the case of positive *Legionella*-containing water samples. In addition, the lyophilization of the LAMP reagents was achieved in the presence of D-trehalose/BSA to allow the prolonged (more than 3 months) storage of these reagents at room temperature and their use outside analytical laboratories, i.e., at the point of need or in resource-limited settings. Despite the fact that the filtering step and the reagent injections are performed manually, they are indeed easy to be performed by non-skilled personnel, allowing, in addition to lyophilization, ease of usage for the proposed chip at the point of need. 

Furthermore, the method presented herein for the detection of *Legionella* was validated with the reference method ISO 11731:2017 providing the same POS/NEG results and was found to be in full agreement for all the water types and concentration levels tested. It showed the highest sensitivity for drinking water (100%) and the lowest for non-drinking water (66.7%). The recommended time for the LAMP reaction is 60 min as it is appropriate for both negative and positive samples, even at concentrations around the legislation limit.

In addition to the color code (Figure 8) for semiquantitative results based on end-point color detection, a computational image analysis was also developed. From the HSV-based methodology, we conclude the following: (1) the samples with different concentrations are numerically separated based on the hue color value; (2) the positive and negative samples are separated clearly; (3) the positive samples with concentrations in the range of 100–1000 CFU/100 mL and greater than 1000 CFU/100 mL are well separated; and (4) the negative samples show hue color values very close to the negative control ones. The chip operation as it was presented in this work is intended for semiquantitative (POS/NEG, based on a color code and computational image analysis) water sample analysis. However, quantitative analysis is also possible through the same computational methodology, considering the quantification of the color change as a function of time during the amplification of different *L. pneumophila* sample concentrations and of the negative control channels. Such computational work is currently in progress.

The presented chip design includes several innovations. As was shown, the microchannel depth was maximized to maximize absorption and, thus, provide high-contrast colorimetric detection, while at the same time keeping the capturing efficiency of the chip (~80%) reasonably high and the microchannel volume at 25 μL, requiring a reasonably low reagent volume and operation cost. In addition, the chip footprint allows the integration of several microchannels on a 5 × 8 cm^2^ PMMA plate, thus allowing the parallel testing of up to five different water samples on the same plate, further reducing the analysis cost. 

It is worth highlighting that the chip presented herein is highly specific for the detection of viable *L. pneumophila* bacteria due to their antibody immunocapturing and specific DNA amplification. Further, the chip was tested for the detection of *Legionella* spp., which was successfully demonstrated. In the future, the chip can be used to detect and discriminate between various *Legionella* serotypes, e.g., *L. pneumophila* serogroups 1–13, *L. longbeachae*, and *L. bozemanii*. In addition, with the appropriate selection of specific antibodies and primers, the chip can be used for the detection of other bacteria contaminating water samples. In this work, in addition to *Legionella*, the on-chip preconcentration and capturing of *Salmonella* bacteria were demonstrated.

## 5. Conclusions

A simple, compact chip was presented for the rapid detection of *Legionella pneumophila* in water samples, integrating for the first time on-chip sample preparation (by bacteria capture, lysis, and LAMP-based amplification of bacterial DNA) with naked-eye or image analysis-based semiquantitative end-point detection, reducing the analysis time required by the gold-standard method by 99%. Compared to PCR, the LAMP-based method presented herein demonstrates many advantages. Although the reduction in analysis time is not as impressive as that of the gold standard method, the LAMP assay exhibits higher sensitivity and specificity than PCR and higher robustness in the presence of certain environmental compounds that often cause the inhibition of PCR, and it enables performing the test in the absence of any sophisticated equipment, allowing its use at the point of need. In fact, the chip features double specificity, combining bacteria capturing on specific antibodies and LAMP-specific primers. In addition, it enables naked-eye qualitative or image analysis-based semiquantitative end-point detection thanks to an optimized microchannel depth, and exhibits high sensitivity and reproducibility, user-friendliness, and long-term storage (18 months) thanks to the lyophilization of antibodies immobilized on the plasma-enhanced surface area of the microfluidic channel. Its analytical performance was validated with the reference method ISO 11731:2017 (gold standard), and it was found to be highly sensitive (100–86.6%) for the analysis of drinking and drilling water, as well as sensitive enough (100–66.7%) for the analysis of non-drinking water (depending on the bacterial contamination level of water). Given the detection limit achieved (50–100 CFU/100 mL), the short analysis time, and the aforementioned advantages of the chip, the proposed platform can have a huge impact in tackling *Legionella* outbreaks and generally find widespread application for fast and reliable waterborne-bacteria detection. 

## 6. Patents

A patent entitled “Diagnostic chip for analyzing the presence of bacteria in a sample”, Patent No GR1010186, has been awarded by the Greek Industrial Property Organization (OBI).

## Figures and Tables

**Table 1:** Sensitivity of Legionella detection for drinking water, non-drinking water, and drilling water spiked with known Legionella concentrations at 3 different levels.

Type of Water	Zero-Level (L_0_) Sensitivity	Medium-Level (L_1_) Sensitivity	High-Level (L_2_) Sensitivity
Drinking	100% [99.5 to 100]	100% [99.5 to 100]	100% [99.5 to 100]
Drilling	100% [99.5 to 100]	86.6% [85.6 to 87.6]	86.6% [85.6 to 87.6]
Non-drinking	100% [99.5 to 100]	66.7% [64.14 to 69.2]	80% [77.47 to 82.53]

## Data Availability

Data access is restricted to protect confidential or proprietary information. However, the data will be available upon request, with permission for the purposes of peer review.

## Supplementary Materials

The following supporting information can be downloaded at https://www.mdpi.com/article/10.3390/bios14050228/s1: 3 types of water samples (i.e., drinking water, non-drinking water, and drilling water) were used with known contamination concentrations at 3 different levels: zero level (L_0_, do not contain Legionella), medium level (L_1_ > 1–2 × 10^2^ CFU/100 mL), and high level (L_2_ > 2 × 10^3^ CFU/100 mL). In each case, 15 repetitive analyses were performed. Representative images of the results are shown in Table S1. Table S1: Representative images are shown for each water type, spiked with Legionella concentrations at three levels.
