# Supplementary material for: A Diagnostic Chip for the Colorimetric Detection of Legionella pneumophila in Less than 3 h at the Point of Need
Source: Biosensors (Basel). 2024 May 4;14(5):228. doi: 10.3390/bios14050228 (PMC11118137; doi:10.3390/bios14050228)
Supplement: Supplementary file 1 [file biosensors-14-00228-s001.zip › biosensors-2963230-supplementary.pdf]

Supplementary Material

A Diagnostic Chip for the Colorimetric Detection of *Legionella pneumophila* in Less than 3 h at the Point of Need

Katerina Tsougeni [1], Anastasia Kanioura [1], Athina S. Kastania [1,2], Kosmas Ellinas [1,†], Antonios Stellas [3], Vassilios Constantoudis [2,3], Galatios Moschonas [4], Nikolaos D. Andritsos [4,‡], Manolis Velonakis [4], Panagiota S. Petrou [1,2], Sotirios E. Kakabakos [1,2], Evangelos Gogolides [1,2] and Angeliki Tserepi [1,2,*]

3 types of water samples (i.e. drinking water, non-drinking water and drilling water) were used with known contamination concentration at 3 different levels: zero level (L_0, do not contain Legionella), medium level ($L_1 > 1\text{-}2 \times 10^2$ CFU/100 mL), and high level ($L_2 > 2 \times 10^3$ CFU/100 mL). In each case, 15 repetitive analyses were performed. Representative images of the results are shown in Table S1.

Table S1: Representative images are shown (out of 15) for each water type, spiked with *Legionella* concentration at three levels

Sample	Zero level (L_0)	Medium level (L_1)	High level (L_2)
Drinking water	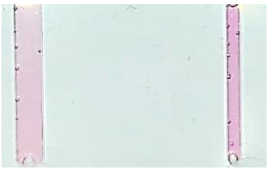	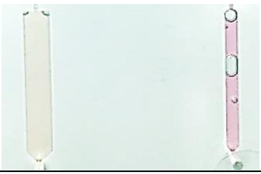	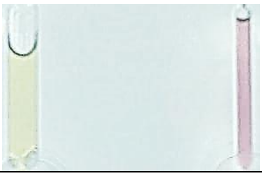
Drilling water	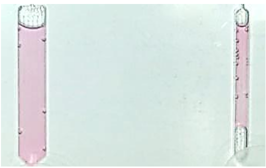	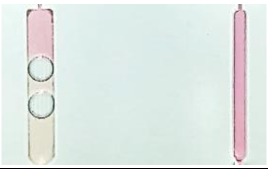	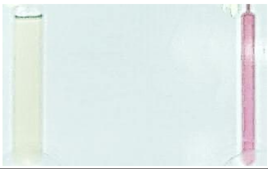
Non-drinking water	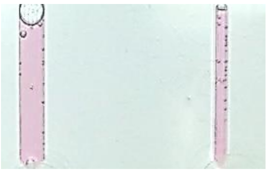	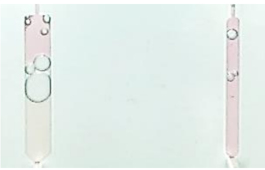	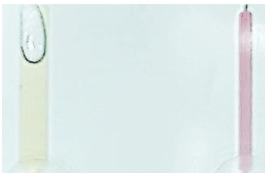
